# Environmental Predictors of Schistosomiasis Persistent Hotspots following Mass Treatment with Praziquantel

**DOI:** 10.4269/ajtmh.19-0658

**Published:** 2019-12-30

**Authors:** Joseph W. Walker, Nupur Kittur, Sue Binder, Jennifer D. Castleman, John M. Drake, Carl H. Campbell, Charles H. King, Daniel G. Colley

**Affiliations:** 1 Center for the Ecology of Infectious Disease, University of Georgia, Athens, Georgia;; 2 University of Georgia College of Public Health, Athens, Georgia;; 3 Schistosomiasis Consortium for Operational Research and Evaluation (SCORE), Center for Tropical and Emerging Global Diseases (CTEGD), University of Georgia, Athens, Georgia;; 4 Odum School of Ecology, University of Georgia, Athens, Georgia;; 5 Center for Global Health and Diseases, Case Western Reserve University School of Medicine, Cleveland, Ohio;; 6 Department of Microbiology, University of Georgia, Athens, Georgia

## Abstract

Schistosomiasis control programs rely heavily on mass drug administration (MDA) campaigns with praziquantel for preventative chemotherapy. Areas where the prevalence and/or intensity of schistosomiasis infection remains high even after several rounds of treatment, termed “persistent hotspots” (PHSs), have been identified in trials of MDA effectiveness conducted by the Schistosomiasis Consortium for Operational Research and Evaluation (SCORE) in Kenya, Mozambique, Tanzania, and Côte d’Ivoire. In this analysis, we apply a previously developed set of criteria to classify the PHS status of 531 study villages from five SCORE trials. We then fit logistic regression models to data from SCORE and publically available georeferenced datasets to evaluate the influence of local environmental and population features, pre-intervention infection burden, and treatment scheduling on PHS status in each trial. The frequency of PHS in individual trials ranged from 35.3% to 71.6% in study villages. Significant relationships between PHS status and MDA frequency, distance to freshwater, rainfall, baseline schistosomiasis burden, elevation, land cover type, and village remoteness were each observed in at least one trial, although the strength and direction of these relationships was not always consistent among study sites. These findings suggest that PHSs are driven in part by environmental conditions that modify the risk and frequency of reinfection.

## INTRODUCTION

Schistosomiasis is a parasitic disease caused by infection with trematode flukes of the genus *Schistosoma* and is estimated to affect more than 200 million people globally.^1^ Human infection occurs through contact with freshwater sources containing live cercariae, which enter the body by penetrating the skin. The transmission cycle continues when eggs excreted in the urine or stool of infected humans enter a fresh water source, hatch, and penetrate a permissive snail host, where they develop into cercariae before being released into the aquatic environment. Chronic egg-induced inflammation due to eggs retained in the tissues of the human host often result in morbidity, with sites of lesions varying between *Schistosoma* species^1^; *Schistosoma haematobium* primarily affects the urogenital system, whereas intestinal schistosomiasis is associated with *Schistosoma mansoni* and *Schistosoma japonicum*. Repeated childhood infections contribute significantly to anemia, undernutrition, and impaired physical and cognitive development.^2–5^

Schistosomiasis control programs can include improving access to clean water and sanitation,^6^ reducing populations of snail hosts,^7^ and implementing campaigns of mass drug administration (MDA) with praziquantel (PZQ) for preventive chemotherapy, with MDA being the most widely used strategy.^1^ In areas of active transmission, the WHO recommends that school-aged children receive MDA with PZQ at different intervals depending on the prevalence of infection in that population, with MDA for adults also recommended under certain conditions.^8^ In 2017, 81.8 million school-aged children and 16.9 million adults received preventative therapy with PZQ, representing 68% of WHO’s Neglected Tropical Disease (NTD) Roadmap coverage target.^9^

Although MDA with PZQ reduces the average prevalence and intensity of *Schistosoma* infection, areas where the schistosomiasis burden remains high despite repeated rounds of MDA, known as “persistent hotspots” (PHSs), have been documented in multiple settings.^10–13^ Possible causes of PHSs include insufficient treatment coverage, reduced rates of infection cure (or egg count reduction) among those treated,^13,14^ sustained transmission within and from untreated populations, environmental conditions, and socioeconomic conditions that lead to frequent human contact with contaminated water sources.^15–17^ A better understanding of how these and other factors shape PHS risk would enable the more efficient allocation of interventions for schistosomiasis control.

A number of studies have used statistical models to quantify the impact of environmental and socioeconomic factors on the prevalence of human schistosomiasis in the absence of MDA.^18–22^ Findings from these analyses, which were conducted across a variety of geographic scales and settings, suggest that prevalence is negatively associated with the distance to freshwater, altitude, and barren land cover, although the effects of temperature and rainfall on prevalence are complex, nonlinear, and mediated by the species of both the *Schistosoma* parasite and its specific snail host. By successfully identifying correlates of schistosomiasis transmission and the areas capable of supporting it, these studies demonstrate the potential of ecological assessments that link population-level measures of infection with open-source georeferenced datasets. However, we are not aware of studies that have attempted to statistically model potential environmental drivers of PHSs.

Between 2012 and 2016, the Schistosomiasis Consortium for Operational Research and Evaluation (SCORE) implemented multiyear cluster randomized trials of MDA regimens with PZQ in five African countries.^23^ In these trials, villages were randomly assigned to receive one of several MDA regimens, which varied in terms of the number of MDAs received during the study period and whether treatments were school-based or community-wide. The prevalence and intensity of infection in each village were assessed before each MDA during the study period, and early analyses revealed variation in the response of these measures to MDA within trials.^10,11^

Kittur et al. evaluated several definitions of PHSs in an analysis based on the first 3 years of SCORE data from Kenya and Tanzania and subsequently applied one of these definitions to describe the frequency and distribution of PHSs in the SCORE study.^11,24^ Using this definition, we combined data from the SCORE studies with publicly available data related to geography, human population patterns, and climate to model characteristics that distinguish PHS villages from responders.

## MATERIALS AND METHODS

### Schistosomiasis Consortium for Operational Research and Evaluation field studies.

This analysis uses data from five field trials of mass PZQ administration for schistosomiasis prevention and control. These studies were carried out by SCORE in association with local partners, and are described in greater detail elsewhere.^23^ Three studies, based in Tanzania, Kenya, and Mozambique, implemented the gaining control of the schistosomiasis protocol, in which ∼150 villages with a *Schistosoma* prevalence ≥25% in an eligibility screening sample of 13- to 14-year-olds were randomly assigned to one of six treatment arms corresponding to different schedules of community-wide therapy, school-based therapy (SBT), and drug holidays (Table 1). Two additional trials, based in Kenya and Côte d’Ivoire, implemented the sustaining control of the schistosomiasis protocol, in which 75 villages with a *S. mansoni* prevalence between 10% and 24% in an eligibility sample based on 13- to 14-year-olds were randomly assigned to one of three study arms. In all trials, data on the prevalence and intensity of *Schistosoma* infection in a sample of 9- to 12-year-olds were collected before each treatment round and in the final year of the trial. With the exception of the Mozambique gaining control study, which targeted *S. haematobium*, *S. mansoni* was the *Schistosoma* species of interest in the other four trials. For trials with *S. mansoni* as the target species, three consecutive daily stool samples were collected from each child and analyzed for egg counts using the duplicate Kato–Katz technique.^25^ For the Mozambique gaining control of *S. haematobium* trial, a single mid-day urine specimen was taken from each child, separated into two 10-mL samples, and filtered. The two filters were finally analyzed for egg counts under microscopy by two different technicians.^26^ The presence and density of eggs in these samples were used to calculate the prevalence and intensity (per gram of stool for *S. mansoni*, or per 10-mL of urine for *S. haematobium*) of schistosomiasis in the study population at each village. After the MDA trial was completed, final treatment was provided to all study participants found to be infected during the year 5 round of data collection. Participants testing positive in prior years received treatment through the trials’ scheduled school-based or community-wide MDA delivery (Table 1). Cleaned village-level data from each of the SCORE studies, including GPS coordinates and annual measures of prevalence and intensity among 9- to 12-year-old children, were put into a standardized datasets and provided for use in this study. These datasets will be released in the future through the ClinEpiDB database.

**Table 1:** Treatment arms of the Schistosomiasis Consortium for Operational Research and Evaluation gaining control and sustaining control of schistosomiasis trials

Study protocol	Treatment arm	Year 1	Year 2	Year 3	Year 4	Year 5
Gaining control	Arm 1	Data collection, CWT	Data collection, CWT	Data collection, CWT	Data collection, CWT	Data collection
	Arm 2	Data collection, CWT	Data collection, CWT	Data collection, SBT	Data collection, SBT	Data collection
	Arm 3	Data collection, CWT	Data collection, CWT	Drug holiday	Drug holiday	Data collection
	Arm 4	Data collection, SBT	Data collection, SBT	Data collection,SBT	Data collection,SBT	Data collection
	Arm 5	Data collection, SBT	Data collection, SBT	Drug holiday	Drug holiday	Data collection
	Arm 6	Data collection, SBT	Drug holiday	Data collection, SBT	Drug holiday	Data collection
Sustaining control	Arm 1	Data collection, SBT	Data collection, SBT	Data collection, SBT	Data collection, SBT	Data collection
	Arm 2	Data collection, SBT	Data collection, SBT	Drug holiday	Drug holiday	Data collection
	Arm 3	Data collection, SBT	Drug holiday	Data collection, SBT	Drug holiday	Data collection

CWT = community-wide therapy; SBT = school-based therapy.

### Persistent Hotspot Status and Predictor Data

For the purpose of this analysis, villages were classified as PHSs if, over the study period, prevalence declined < 35% relative to the value observed in year 1 and/or intensity declined < 50%. Villages were classified as “responders” if a decline exceeding the threshold was observed in both measures.^24^

To supplement SCORE data on different treatment schedules and baseline infection prevalence and intensity, we derived data on a number of geographic and environmental features for each village, which are described in Table 2. All data-processing and subsequent statistical analysis were conducted in version 3.5.1 of the R software environment. Spatial data were handled using the “raster” package. We derived two distinct precipitation products, absolute and relative rainfall over the study period, using the National Oceanic and Atmospheric Administration-produced Africa Rainfall Climatology 2.0 dataset, which uses satellite and weather station data to provide daily rainfall estimates over the African continent at 0.1 decimal degree resolution from 1983 to the present.^27^ For absolute rainfall, we calculated the average 3-month rainfall over the study period for January–March, April–June, July–September, and October–December. To derive these predictors, we extracted the estimated daily rainfall at the location of each study village for each day in 2012–2016, summed to calculate the total rainfall within each consecutive 3-month period of each year, and averaged across years. Meanwhile, for relative rainfall, we calculated the median *Z*-score of 3-month rainfall over the study period from a historic baseline. For each 3-month period, a time series of trimonthly rainfall between 1983 and 2018 (36 observations, one annually) was derived at each village location. At each village, the *Z*-scores of observations during study years (2012–2016) were then derived and summarized by the median. These measures of how extreme rainfall levels were during the study period, relative to historic patterns at the same location, replaced absolute rainfall variables in supplementary models.

**Table 2:** Village-level enviro-geographic, baseline epidemiological, and treatment features

Predictor	Source	Data processing
Treatment arm	Schistosomiasis Consortium for Operational Research and Evaluation standardized dataset	
Year 1 prevalence (%)		
Year 1 infection intensity (mean eggs per gram of stool (*S. mansoni*) or 10-mL urine (*S. mansoni*)		
Distance to freshwater (km)	Kummu et al.^28^	Mean value within 1 km of village coordinates computed
Travel time to a major* urban center (hours)	Weiss et al.^29^	Mean value within 1 km of village coordinates computed
Population density (population per km^2^)	WorldPop^30^	Mean value within 1 km of village coordinates computed
Agricultural land cover (%)	GlobCover^31^	Value within 1 km of village coordinates computed
Altitude (m)	NASA SRTM^32^	Mean value within 1 km of village coordinates computed
Absolute rainfall: average 3-month rainfall (mm), 2012–2016	Africa Rainfall Climatology (ARC) 2.0^27^	Methods described in text. Spatially accurate within 0.1 decimal degrees, the resolution of the rainfall dataset
Relative rainfall: median *Z*-score of 3-month rainfall from historical baseline, 2012–2016	ARC 2.0^27^	Methods described in text. Spatially accurate within 0.1 decimal degrees, the resolution of the rainfall dataset

*S. mansoni* = *Schistosoma mansoni.*

* Population of 50,000 or greater.

### Statistical analysis.

Using the predictor variables described earlier and PHS status as the binary response, we fit one multivariate and several univariate fixed-effect logistic regression models to data from each of the five trials. To avoid potentially unrealistic assumptions of linearity, all numeric predictor variables were categorized into bins based on median and tercile values within each of the five sets of villages (one for each of the five trials) before model fitting. To assess the association between predictor variables and village PHS status, odds ratios and corresponding 95% CIs were extracted from each logistic regression model. The threshold used for statistical significance was set at *P <* 0.05 in all cases unless stated otherwise.

## RESULTS

### Observed frequency of persistent hotspots.

In the gaining control trials, the observed frequency of PHS villages ranged from 35% in the Kenya study to 72% in the Tanzania study (Table 3). In the sustaining control trials, 45% and 55% of villages were classified as PHSs at the Côte d’Ivoire and Kenya study sites, respectively. The proportion of villages classified as PHSs was significantly lower (*P* < 0.0001) in the Kenya gaining control trial (35%) than in the Tanzania and Mozambique gaining control trials (72% and 65%, respectively). Differences in the proportion of PHS villages between the Tanzania and Mozambique gaining control trials (72% versus 65%) and between the Kenya and Côte d’Ivoire sustaining control trials (55% versus 45%) were not statistically significant (*P* = 0.32 and *P* = 0.33, respectively).

**Table 3:** Proportion of persistent hotspots in study sites by the trial protocol

Trial protocol	Study site	Target species	Persistent hotspot villages, *n* (%)
Gaining control	Tanzania (148 villages)	*S. mansoni*	106 (72%)
	Kenya (150 villages)	*S. mansoni*	53 (35%)
	Mozambique (133 villages)	*Schistosoma haematobium*	87 (65%)
Sustaining control	Kenya (75 villages)	*S. mansoni*	41 (55%)
	Côte d’Ivoire (75 villages)	*S. mansoni*	34 (45%)

*S. mansoni* = *Schistosoma mansoni.*

### Descriptive analysis.

#### Distance to freshwater.

**Figure 1.**
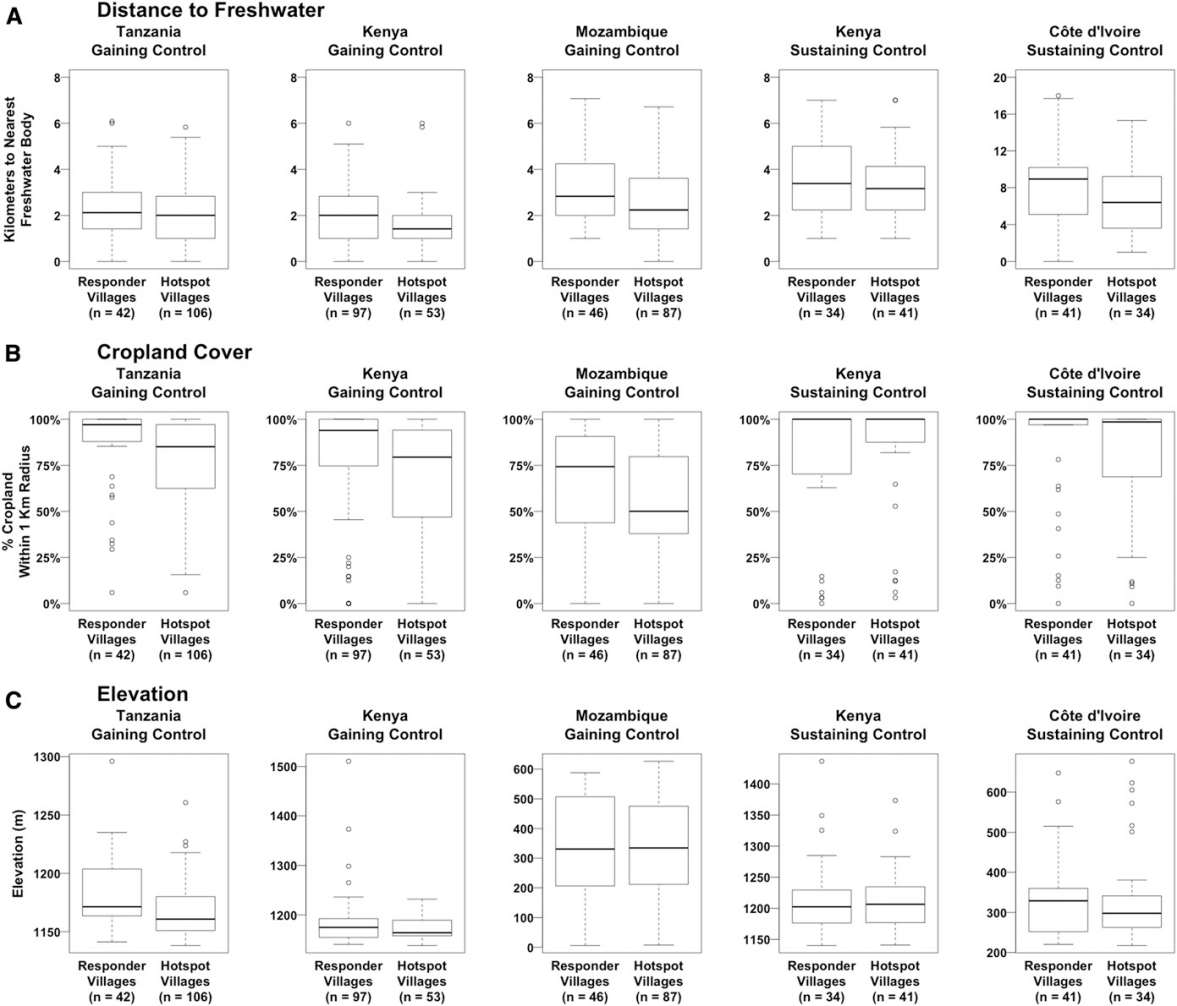
Differences between persistent hotspot and responder villages in their distance to freshwater, cropland cover, and elevation.

In the three gaining control studies and the Kenya sustaining control study, most of the villages were located within 4 km of a freshwater body, whereas villages at the more arid Côte d’Ivoire study site had a median distance to water of 7.3 km, with some villages located up to 18 km from a freshwater body (Figure 1A). At all study sites, the distance to water was generally greater for responder villages than for PHS villages, and this relationship was strongest at the Kenya and Mozambique gaining control study sites.

#### Cropland cover.

A high proportion of the land around study villages was frequently devoted to agriculture, with the median percentage of the area within 1 km of villages classified as cropland ranging from 58.3% in the Mozambique gaining control study to 100% in the Kenya and Côte d’Ivoire sustaining control studies (Figure 1B). Persistent hotspot villages appeared to be associated with lower levels of cropland relative to responder villages at all study sites, with the exception of the Kenya sustaining control trial, for which the opposite relationship was observed.

#### Elevation.

The study site with the most variable terrain in terms of elevation change was the Mozambique gaining control trial, with a difference in altitude of 621 m between the highest and lowest study village (Figure 1C). Boxplots of the elevation of study villages in the Tanzania and Kenya gaining control trials indicate that PHS villages are somewhat more low-lying than responder villages. It is likely that the lower elevation study villages at these sites were located closer to the shores of Lake Victoria, the largest freshwater body in the area (Supplemental Figures 2 and 3).

#### Population density.

**Figure 2.**
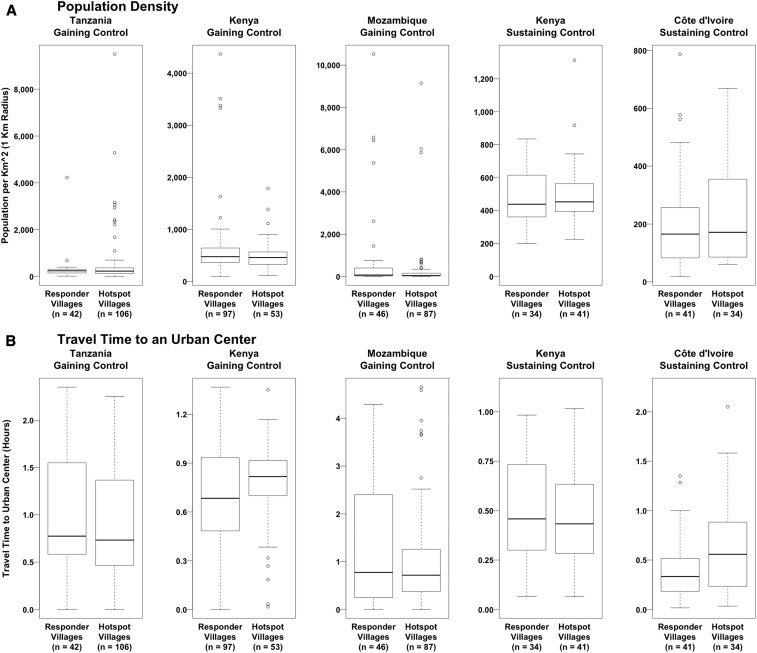
Differences between persistent hotspot and responder villages in their population density and travel time to an urban center.

Within a 1-km-radius buffer zone, most villages contained fewer than 600 people per square kilometer, with a handful of study villages in the urban areas of the gaining control study sites having a population density over 2,000 people per square kilometer (Figure 2A). The village with the highest population density, with 10,521 people per square kilometer, was observed in the Mozambique gaining control study site. The median population density of PHS and responder villages was comparable at all study sites.

#### Travel time to an urban center.

Between study sites, the median travel time from study villages to an urban center, defined as a settlement of more than 50,000 people, ranged from 24 to 46.2 minutes (Figure 2B). In the Kenya gaining control and Côte d’Ivoire sustaining control studies, PHS villages appear to be somewhat more remote from urban settlements than responder villages, although the travel time in both studies was less than an hour for the overwhelming majority of villages.

#### Rainfall.

**Figure 3.**
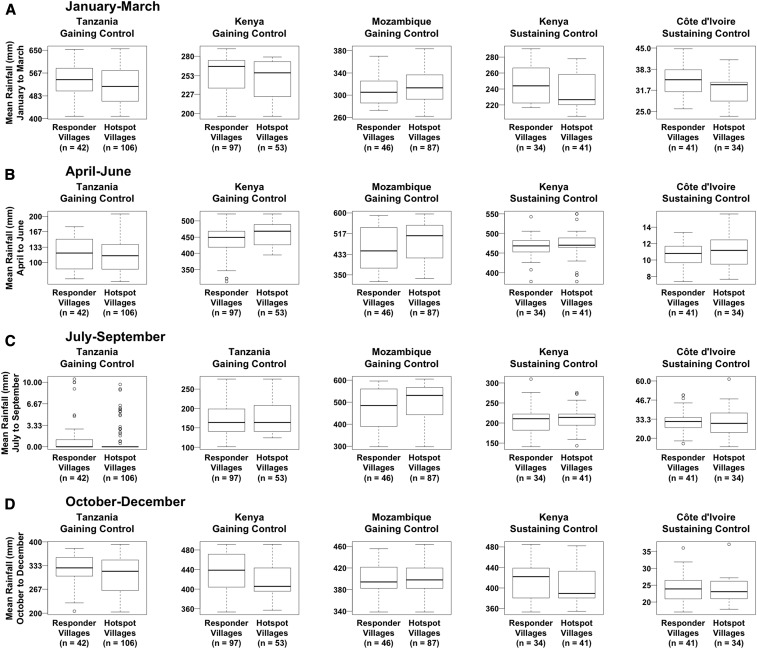
Differences between persistent hotspot and responder villages in absolute trimonthly rainfall.

Absolute rainfall varied in seasonal patterns over the study period at each site, with precipitation peaking between October and March at the Tanzania gaining control site, between April and September at the Mozambique gaining control site, between July and September at the Côte d’Ivoire sustaining control site, and twice annually, between April and June and between October and December, at the Kenya gaining and sustaining control study sites (Figure 3). The ratio of the median of the average trimonthly rainfall in PHS to responder villages ranged from 0.9 in the October–December period of the Kenya sustaining control study to 1.1 in the April–June period of the Mozambique gaining control study. Meanwhile, the differences in the median values of average trimonthly rainfall between PHS and responder villages ranged from 33 mm higher for responder villages between October and December of the Kenya gaining control study to 61 mm higher for PHS villages between April and June in the Mozambique gaining control study.

**Figure 4.**
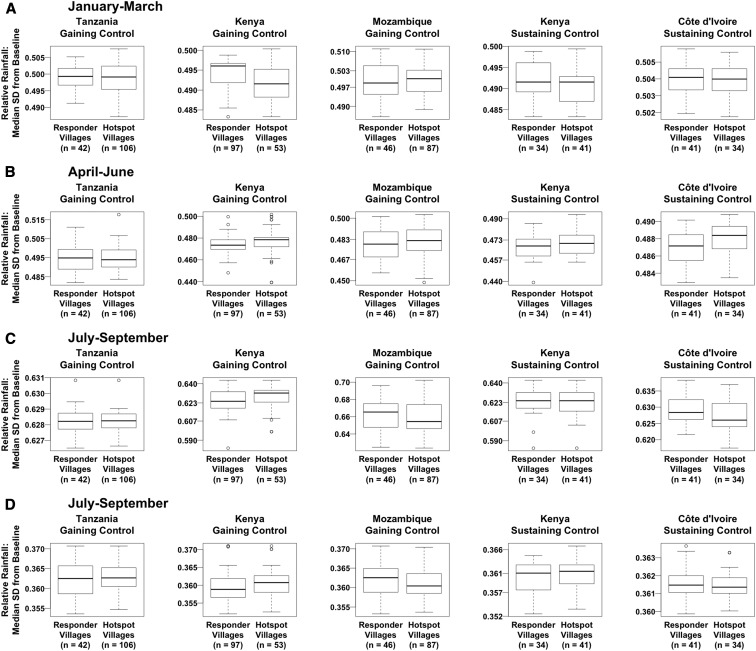
Differences between persistent hotspot and responder villages in relative trimonthly rainfall.

The median SD of rainfall during the study period from the historical baseline was positive for each study village in all trimonthly periods, indicating that rainfall was higher than the historical baseline in at least half of the years of the study period (Figure 4). The greatest median SD from baseline, 0.7, was observed in the July–September period of the Mozambique gaining control study. Differences in relative rainfall between villages were small, with no two villages having median SDs from baseline that differed by more than 0.1 in any 3-month period.

Mean monthly rainfall was not considered as a predictor of PHS status in the Tanzania gaining control trial for the months of June, July, August, and September, or in the Côte d’Ivoire sustaining control trial for the month of June because variation in precipitation was highly limited: in each of these months, at least 78% of villages received no rainfall at all and no village received over 11 mm of rainfall (averaged over the observation period) in a single month.

### Regression analysis.

Tables 4 and 5 display the statistically significant outputs from fixed-effect models fit to individual gaining control and sustaining control trials, respectively. Unabridged model outputs, including odds ratio estimates that were not statistically significant at *P* < 0.05 in any model, are shown in Supplemental Tables 1 and 2

**Table 4:** Significant relationships between persistent hotspot status and predictors in individual gaining control trials

Predictor	Tanzania trial			Kenya trial			Mozambique trial		
	Predictor value	Univariate OR (95% CI)	Multivariate OR (95% CI)	Predictor value	Univariate OR (95% CI)	Multivariate OR (95% CI)	Predictor value	Univariate OR (95% CI)	Multivariate OR (95% CI)
Treatment arm	Arm 1	1	1	Arm 1	1	1	Arm 1	1	1
	Arm 3	1.80 (0.53–6.49)	2.25 (0.51–10.62)	Arm 3	2.02 (0.63–6.80)	4.42 (0.85–25.09)	Arm 3	2.83 (0.85–10.20)	**4.95** (**1.08–26.33)**
	Arm 4	1.54 (0.47–5.29)	1.49 (0.34–6.73)	Arm 4	1.06 (0.30–3.72)	1.31 (0.26–6.59)	Arm 4	3.20 (0.92–12.42)	**6.15** (**1.19–38.62)**
	Arm 5	1.90 (0.56–6.83)	1.31 (0.28–6.25)	Arm 5	2.37 (0.75–7.99)	3.95 (0.78–22.44)	Arm 5	**3.60** (**1.05–13.86)**	**5.02** (**1.05–27.58)**
Baseline prevalence	< 38%	1	1	< 51.3%	1	1	< 57.14%	1	1
	> 72.9%	2.01 (0.79–5.33)	2.85 (0.46–19.86)	> 75.4%	**3.81** (**1.68–8.94)**	**7.97** (**1.32–56.48)**	> 82.83%	1.07 (0.43–2.64)	2.71 (0.66–12.26)
Baseline intensity (mean eggs per gram or per 10 mL urine)	< 25.15	1	1	< 32.45	1	1	< 33.09	1	1
	25.15–122.50	1.00 (0.42–2.37)	0.80 (0.24–2.59)	32.45–88.81	0.68 (0.27–1.66)	**0.18** (**0.04–0.70)**	33.09–95.00	0.79 (0.31–2.04)	0.51 (0.13–1.90)
	> 122.50	1.40 (0.58–3.45)	0.44 (0.08–2.27)	> 88.81	**2.74** (**1.22–6.35)**	0.28 (0.05–1.52)	> 95.00	**0.35** (**0.14–0.84)**	0.26 (0.06–1.01)
Distance to freshwater (km)	< 1.41	1	1	< 1.00	1	1	< 2	1	1
	> 2.00	0.51 (0.21–1.21)	0.60 (0.18–1.97)	> 2.00	**0.24** (**0.06–0.92)**	1.33 (0.14–13.27)	> 3.00	0.68 (0.26–1.69)	0.34 (0.09–1.21)
Travel time to urban center (hours)	< 0.58	1	1	< 0.57	1	1	< 0.47	1	1
	0.58–1.13	1.11 (0.45–2.72)	0.40 (0.11–1.32)	0.57–0.87	**2.94** (**1.25–7.24)**	**6.25** (**1.15–40.33)**	0.47–1.07	1.36 (0.56–3.37)	0.72 (0.22–2.24)
	> 1.13	0.85 (0.36–2.03)	1.11 (0.25–4.97)	> 0.87	2.12 (0.89–5.24)	**9.86** (**1.21–108.54)**	> 1.07	0.94 (0.40–2.23)	0.70 (0.19–2.43)
% cropland (1 km radius)	< 75.76%	1	1	< 76.69	1	1	< 44.12%	1	1
	> 96.97%	**0.29** (**0.11–0.70)**	0.33 (0.09–1.14)	> 94.29%	**0.31** (**0.13–0.70)**	0.92 (0.17–4.84)	> 77.14%	0.43 (0.17–1.03)	**0.27** (**0.08–0.99)**
Elevation (m)	< 1,155	1	1	< 1,161	1	1	< 271	1	1
	1,155–1,176	**0.20** (**0.07–0.54)**	0.40 (0.11–1.35)	1,161–1,184	0.66 (0.29–1.47)	0.59 (0.14–2.25)	271–430	1.11 (0.45–2.72)	0.47 (0.11–1.87)
	> 1,176	**0.30** (**0.10–0.81)**	0.55 (0.13–2.20)	> 1,184	0.51 (0.22–1.16)	1.15 (0.23–5.97)	> 430	0.85 (0.36–2.03)	0.42 (0.08–1.99)
Mean rainfall (mm), April–June	< 96.59	1	1	< 426.15	1	1	< 434.49	1	1
	96.59–133.43	2.15 (0.86–5.69)	2.07 (0.58–7.79)	426.15–468.15	1.35 (0.55–3.47)	1.42 (0.29–7.36)	434.49–539.74	**2.57** (**1.05–6.54)**	**22.62** (**1.91–692.12)**
	> 133.43	0.94 (0.41–2.18)	1.02 (0.19–5.42)	> 468.15	**3.71** (**1.55–9.42)**	**16.04** (**2.07–158.49)**	> 539.74	2.15 (0.92–5.18)	6.88 (0.23–367.89)
Mean rainfall (mm), October–December	< 303.65	1	1	< 402.56	1	1	< 384.36	1	1
	> 339.81	0.66 (0.25–1.66)	0.78 (0.11–5.65)	> 443.15	**0.33** (**0.14–0.74)**	0.24 (0.05–1.13)	> 411.68	0.97 (0.39–2.36)	1.53 (0.35–6.90)

Odds ratios that are statistically significant at *P* < 0.05 are denoted in bold.

**Table 5:** Relationships between persistent hotspot status and predictors in individual sustaining control trials

Predictor	Kenya trial			Côte d’Ivoire trial		
	Predictor value	Univariate OR (95% CI)	Multivariate OR (95% CI)	Predictor value	Univariate OR (95% CI)	Multivariate OR (95% CI)
Treatment arm	Arm 1	1	1	Arm 1	1	1
	Arm 2	1.64 (0.53–5.21)	2.57 (0.49–15.62)	Arm 2	**3.19 (1.02–10.60)**	**30.73 (3.06–681.47)**
Baseline intensity (mean eggs per gram)	< 8.87	1	1	< 40.74	1	1
	8.87–18.35	**0.21 (0.06–0.68)**	**0.14 (0.02–0.79)**	40.74–81.50	1.38 (0.45–4.26)	3.16 (0.47–27.60)
	> 18.35	**0.29 (0.08–0.94)**	0.40 (0.05–2.77)	> 81.50	0.85 (0.27–2.62)	0.57 (0.04–7.78)
Travel time to urban center (hours)	< 0.37	1	1	< 0.28	1	1
	> 0.62	0.66 (0.21–2.04)	0.65 (0.05–8.69)	> 0.56	**3.54 (1.12–12.04)**	3.70 (0.54–29.31)
% cropland (1 km radius)	< 87.75%	1	1	< 96.94%	1	1
	> 97.06%	1.02 (0.37–2.75)	1.14 (0.09–14.14)	> 97.06%	0.39 (0.14–1.05)	**0.13 (0.01–0.97)**
Elevation (m)	< 1,192	1	1	< 267	1	1
	1,192–1,223	1.62 (0.53–5.09)	**6.07 (1.0004–50.59)**	267–343	1.91 (0.63–6.02)	1.39 (0.15–14.00)
Mean rainfall (mm), January–March	< 222.39	1	1	< 31.37	1	1
	> 256.92	0.48 (0.14–1.56)	2.81 (0.06–162.56)	> 35.01	**0.29 (0.09–0.91)**	**0.05 (0.004–0.34)**

Odds ratios that are statistically significant at *P* < 0.05 are denoted in bold.

In the Tanzania gaining control trial, villages at lower elevation and with a lower proportion of cropland in the surrounding area were significantly more likely to become PHSs in univariate analysis, whereas no significant relationships between predictors and PHS status were detected by a multivariate model. Meanwhile, in the Kenya gaining control trial, PHSs were significantly associated with lower absolute rainfall between October and December, lower cropland cover, and closer proximity to freshwater in univariate models. In both univariate and multivariate models, PHSs were significantly associated with greater travel time to major urban centers, higher prevalence of infection at baseline, and higher absolute rainfall between April and June. Villages with a greater intensity of infection at baseline were significantly more likely to become PHSs in a univariate model, but this relationship was reversed when controlling for other factors in the multivariate model. Finally, in the Mozambique gaining control trial, multivariate models found that the risk of becoming a PHS was significantly greater for villages that received a higher volume of rainfall between April and June, had lower cropland cover, or were assigned to treatment arms 3, 4 (SBT in all 4 years), or five instead of arm 1. No significant relationships between PHS status and treatment arm were detected in the individual Kenya and Tanzania gaining control trials.

Observed relationships between precipitation and PHS status sometimes differed when variables describing the absolute level of rainfall during the study period were replaced by measures of how rainfall during the study period deviated from the location-specific historical baseline (Supplemental Tables 3 and 4) Between January and March, relative rainfall was negatively associated with PHSs in the Kenya gaining control study, whereas higher relative rainfall between April and June was a significant predictor of PHSs in both the Kenya gaining and sustaining control trials and the Côte d’Ivoire sustaining control trial. Between July and September, relative rainfall was positively associated with PHSs in the Kenya gaining control trial, whereas the opposite effect was observed in the Kenya and Côte d’Ivoire sustaining control trials. Between October and December, villages with higher relative rainfall during the study period were significantly less likely to become PHSs in the Mozambique gaining control trial.

## DISCUSSION

Persistent hotspot threaten both the control of morbidity and the elimination of schistosomiasis as a public health problem, which the WHO has advocated that national health programs adopt as incremental program goals since 2012.^33^ This is the first study to evaluate potential factors associated with PHSs of schistosomiasis by analyzing publicly available geographic and environmental data with village-level outcomes data from multiple MDA trials. Unsurprisingly, we found that PHSs were often more likely to arise in villages that experienced MDA less frequently, particularly those that went untreated over 2 consecutive years. This is consistent with a body of evidence indicating that MDA only temporarily affects schistosomiasis transmission when other environmental control measures are not implemented concurrently partially because of the fact that treatment with PZQ does not prevent reinfection or kill immature schistosomes.^34^ We also detected a significant negative relationship between the distance to freshwater and the likelihood of a village becoming a PHS in some models, which aligns with the hypothesis that children living closer to water are exposed to cercariae and become infected more frequently, thus undermining the effectiveness of MDA for controlling schistosomiasis.^15,19,35,36,37^

In each trial, with the exception of the Kenya sustaining control study, we observed PHSs less frequently in villages containing or surrounded by a higher proportion of cropland, as classified by remote sensing. Further review of remote sensing data indicates that most nonagricultural land in the vicinity of study villages took the form of forest, grassland, and surface water (Supplemental Figure 1). The presence of surface water close to a village may facilitate infection when alternative water sources are unavailable.^33,34^ It is important to note that none of the cropland detected in the vicinity of study villages was classified as irrigated or flooded. As areas where humans may frequently come into contact with high-quality snail habitat for extended periods of time, irrigated and flooded agricultural land has been identified as highly conducive to schistosomiasis transmission.^38–40^ The negative relationship we observed between cropland cover and PHS risk at some study sites may be weakened or reversed in settings where agricultural irrigation and flooding is more common.^41,42^ Going forward, the use of higher resolution and more up-to-date remote sensing data could further refine our understanding of how land cover influences PHS: the GlobCover dataset we used is based on the 300-m resolution imagery from 2009, 3 years before the start of the study period.^31^

The effect of rainfall on PHS status in this analysis depended on both the study site and time of year. In both the Kenya and Mozambique gaining control trials, PHSs were associated with higher rainfall between April and June. Meanwhile, low rainfall between January and March and between October and November was predictive of PHSs in the Côte d’Ivoire sustaining control trial and the Kenya gaining control trial, respectively. Continental-scale statistical modeling has failed to characterize a consistent relationship between precipitation and schistosomiasis transmission potential, suggesting that these relationships vary between settings.^18,43^ Higher rainfall can facilitate the transmission of schistosomiasis by moving excreted eggs into freshwater bodies, transporting snails and schistosomes to new locations, and supporting the establishment and persistence of suitable water bodies.^15,44–46^ However, high precipitation levels can also reduce human infection risk when flooding or fast-moving water flows reduce or eliminate snail host populations.^15^ Nevertheless, which, if any, of these mechanisms are responsible for the observed relationships between rainfall and PHSs is unclear.

For the most part, we did not observe major differences in the predictors of *S. mansoni* and *S. haematobium* PHSs in the gaining control studies (Table 4). Treatment arm was the only factor that was significantly associated with PHS status in the one *S. haematobium* gaining control trial, but not in either of the two *S. mansoni* gaining control trials. Meanwhile, no factors were significantly associated with PHS status in both of the *S. mansoni* gaining control studies. Although the intermediate hosts of *S. haematobium* and *S. mansoni*, snails of the genus *Bulinus* and *Biomphalaria*, respectively, both primarily reside in shallow freshwater bodies, *Bulinus* snails are distinguished by being able survive in warmer and more barren environments.^47^ As a result, differences in the frequency and drivers of PHSs between *S. mansoni* and *S. haematobium* may be more pronounced in areas that are hotter and more arid than the sites where the gaining control studies were conducted. We were unable to evaluate possible differences in PHS drivers between *Schistosoma* species in the sustaining control trials, as both of these studies used *S. mansoni* as the target schistosome.

The SCORE studies were implemented with engagement of NTD program managers and with clearly defined targets for coverage. The finding of PHS shows that these efforts will not always be sufficient to control schistosomiasis among children in highly endemic areas and that further efforts, such as snail control, provision of sanitation and safe water, and more frequent or broadly targeted MDA, may be needed in some settings.^48^ The ability to predict PHS early in the implementation of programs based on easily obtainable data could allow NTD program managers to target interventions more quickly to where they will be most needed. We observed associations between PHS status and some environmental factors, but these trends were inconsistent between study sites, in line with the highly focal dynamics of schistosomiasis transmission. Further studies that combine a variety of village-level data with detailed models of local transmission dynamics may help develop predictive tools that can lead to more efficient and effective programs.

## Supplemental tables and figures

Supplemental materials
