# Supplementary material for: Environmental Predictors of Schistosomiasis Persistent Hotspots following Mass Treatment with Praziquantel
Source: Am J Trop Med Hyg. 2019 Dec 30;102(2):328–38. doi: 10.4269/ajtmh.19-0658 (PMC7008331; doi:10.4269/ajtmh.19-0658)
Supplement: Supplementary file 1 [file tpmd190658.SD1.pdf]

Supplementary figure 1 Distributions of Land Cover Types in the Vicinity of Study Villages by Trial

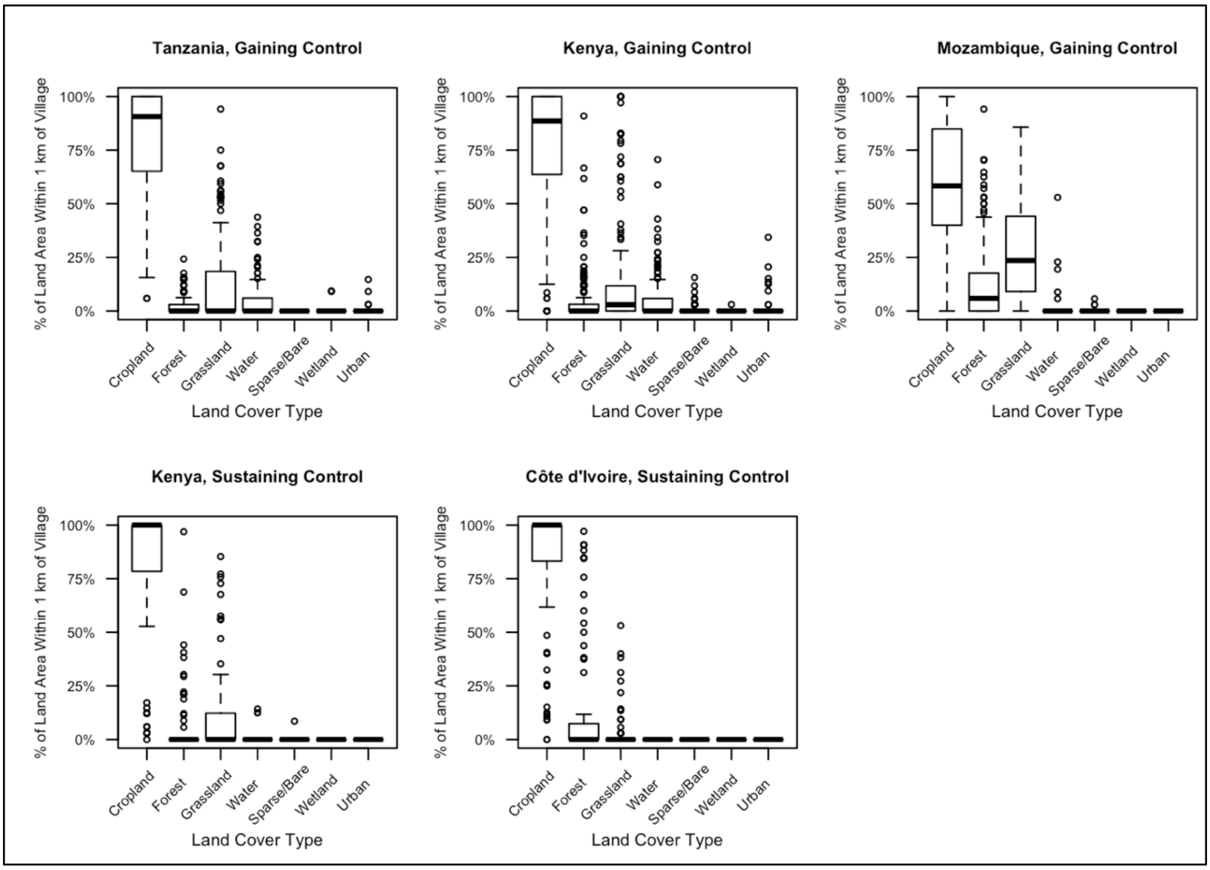

Supplementary Figure 2. Map of the Tanzania Gaining Control Study Site. Red circles and yellow triangles indicate persistent hotspot and responder villages, respectively. Landsat-8 image courtesy of the U.S. Geological Survey.

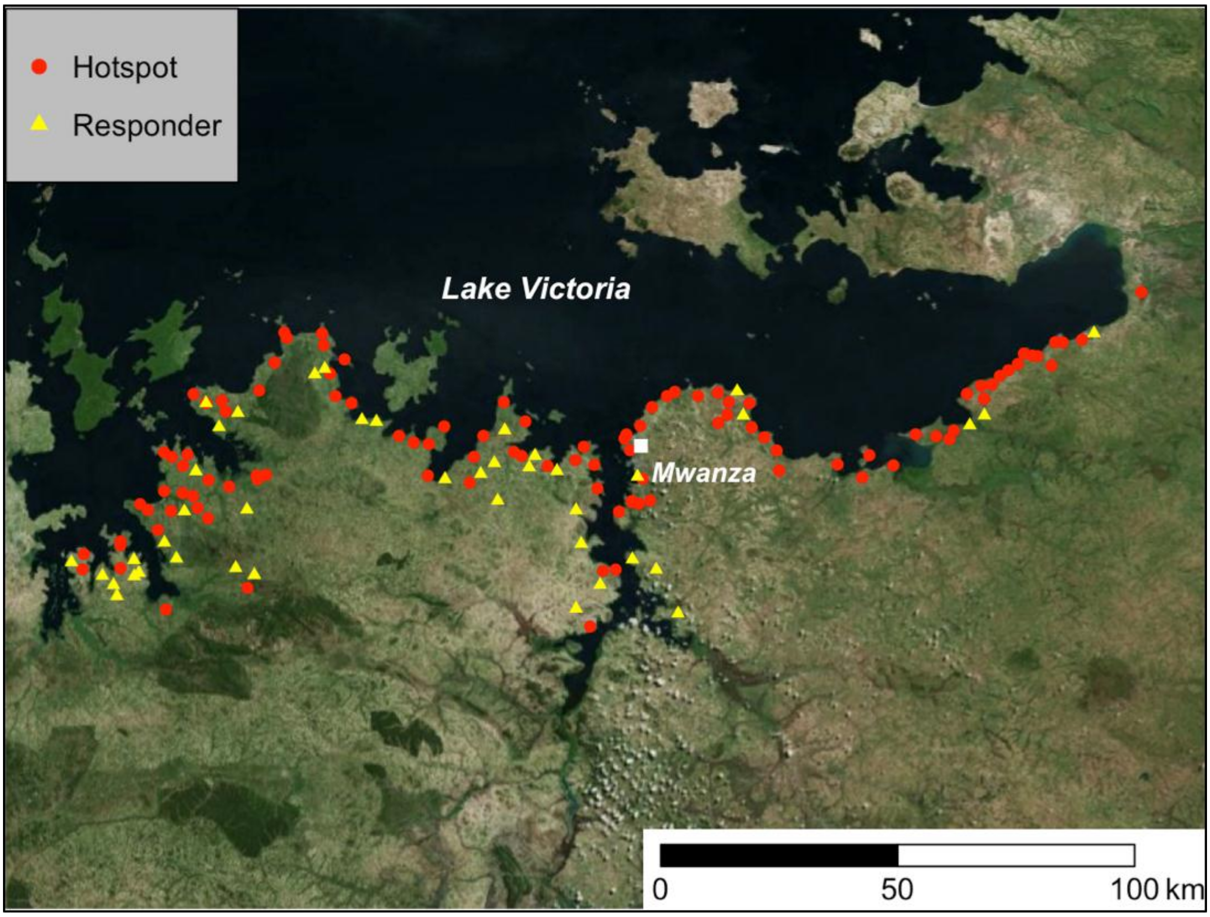

Supplementary Figure 3. Map of the Kenya Gaining Control Study Site. Red circles and yellow triangles indicate persistent hotspot and responder villages, respectively. Landsat-8 image courtesy of the U.S. Geological Survey.

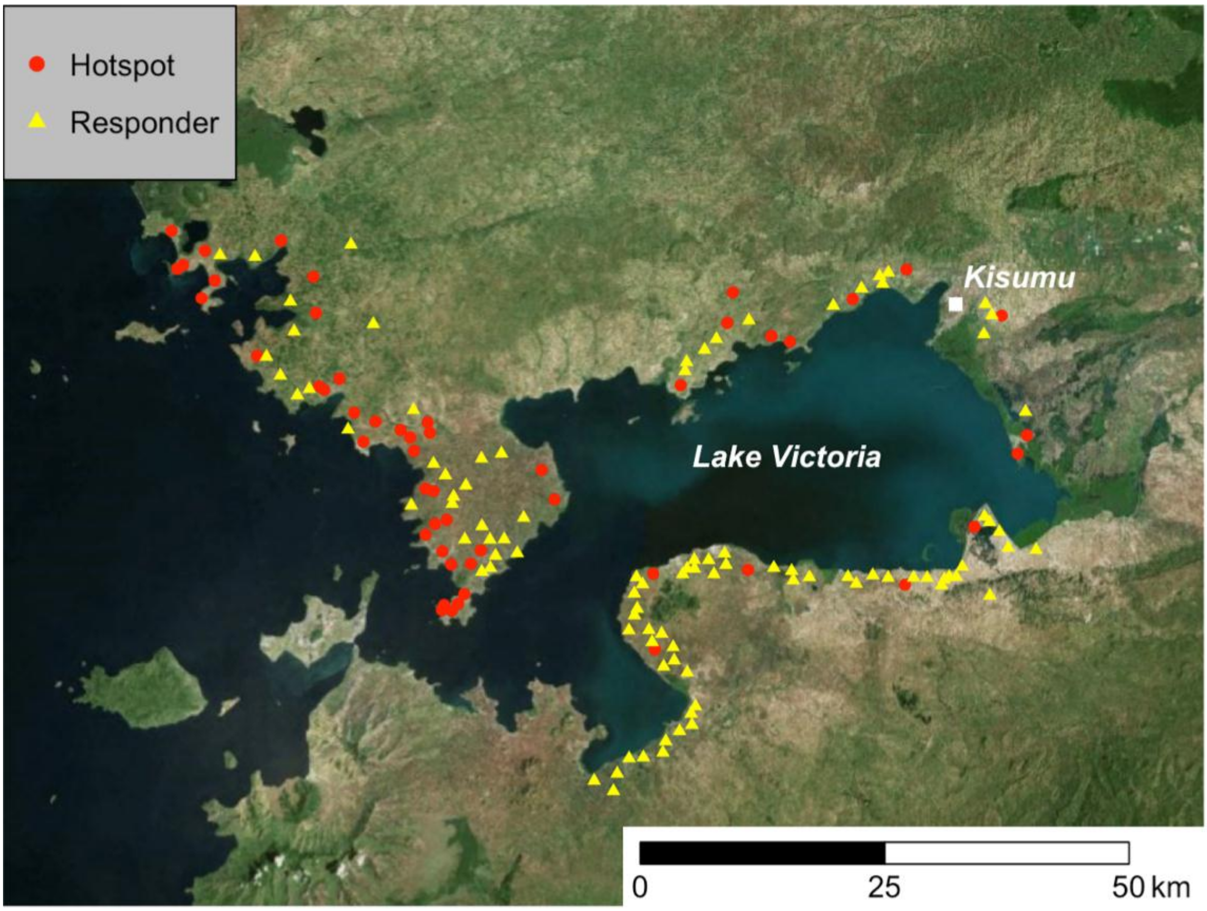

Supplementary Figure 4. Map of the Mozambique Gaining Control Study Site. Red circles and yellow triangles indicate persistent hotspot and responder villages, respectively. Landsat-8 image courtesy of the U.S. Geological Survey.

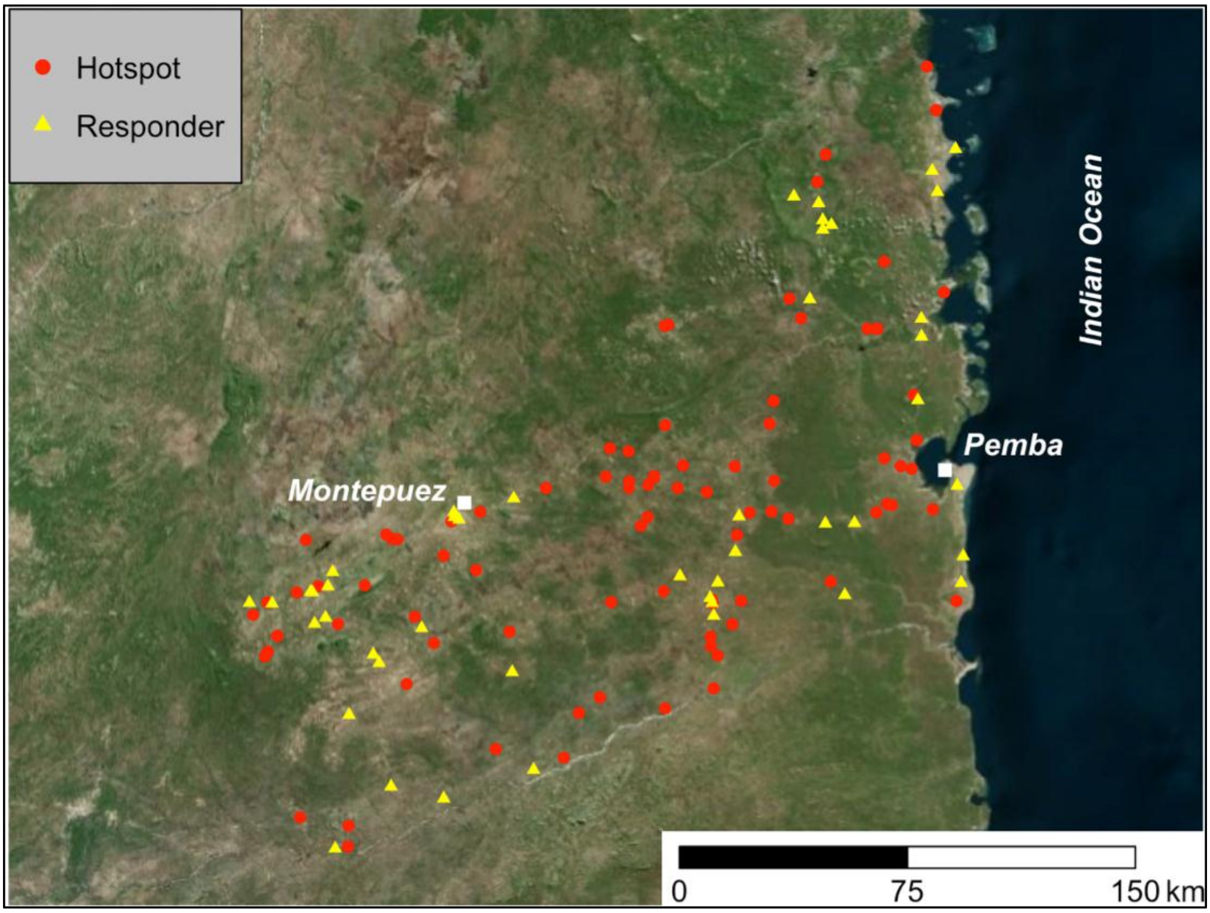

Supplementary Figure 5. Map of the Kenya Sustaining Control Study Site. Red circles and yellow triangles indicate persistent hotspot and responder villages, respectively. Landsat-8 image courtesy of the U.S. Geological Survey.

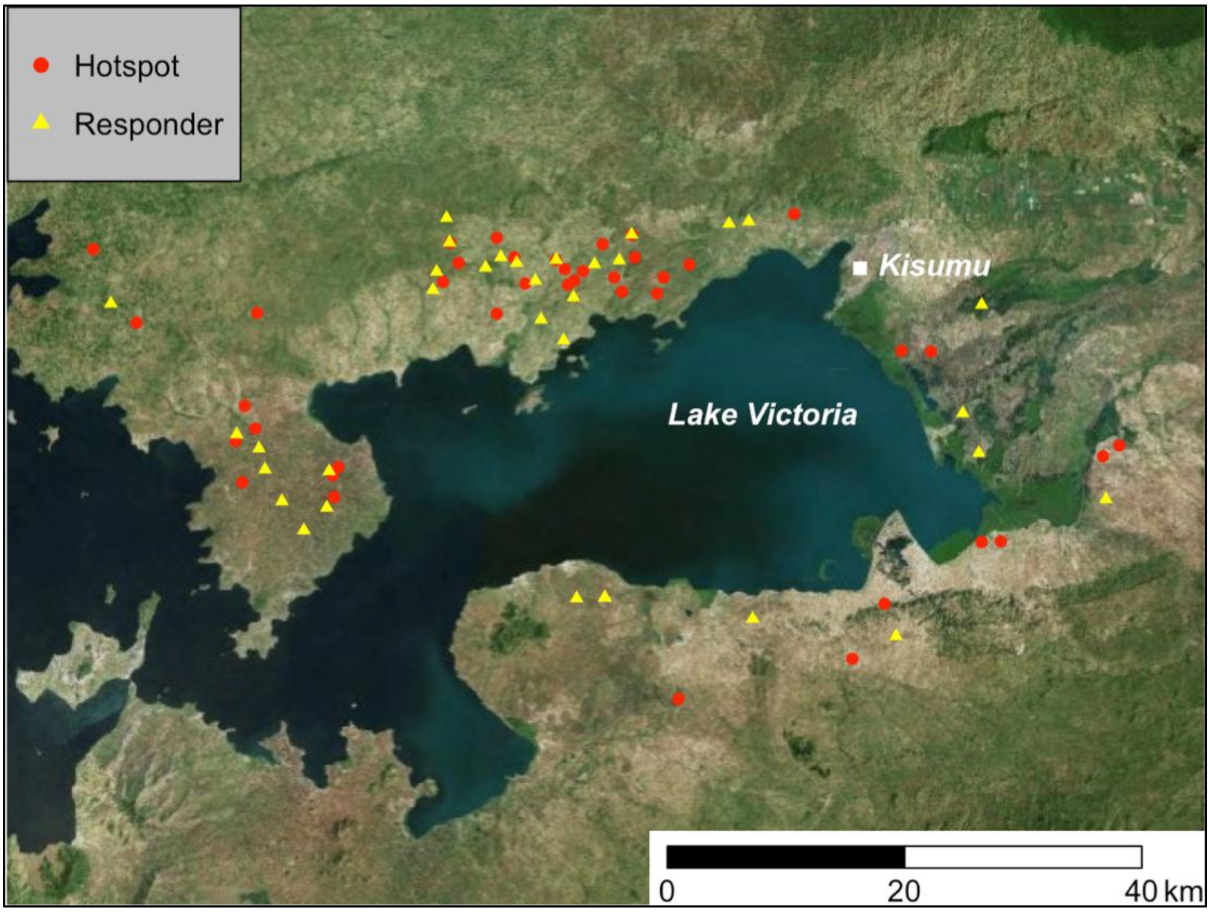

Supplementary Figure 6. Map of the and Côte d'Ivoire Sustaining Control Study Site. Red circles and yellow triangles indicate persistent hotspot and responder villages, respectively. Landsat-8 image courtesy of the U.S. Geological Survey.

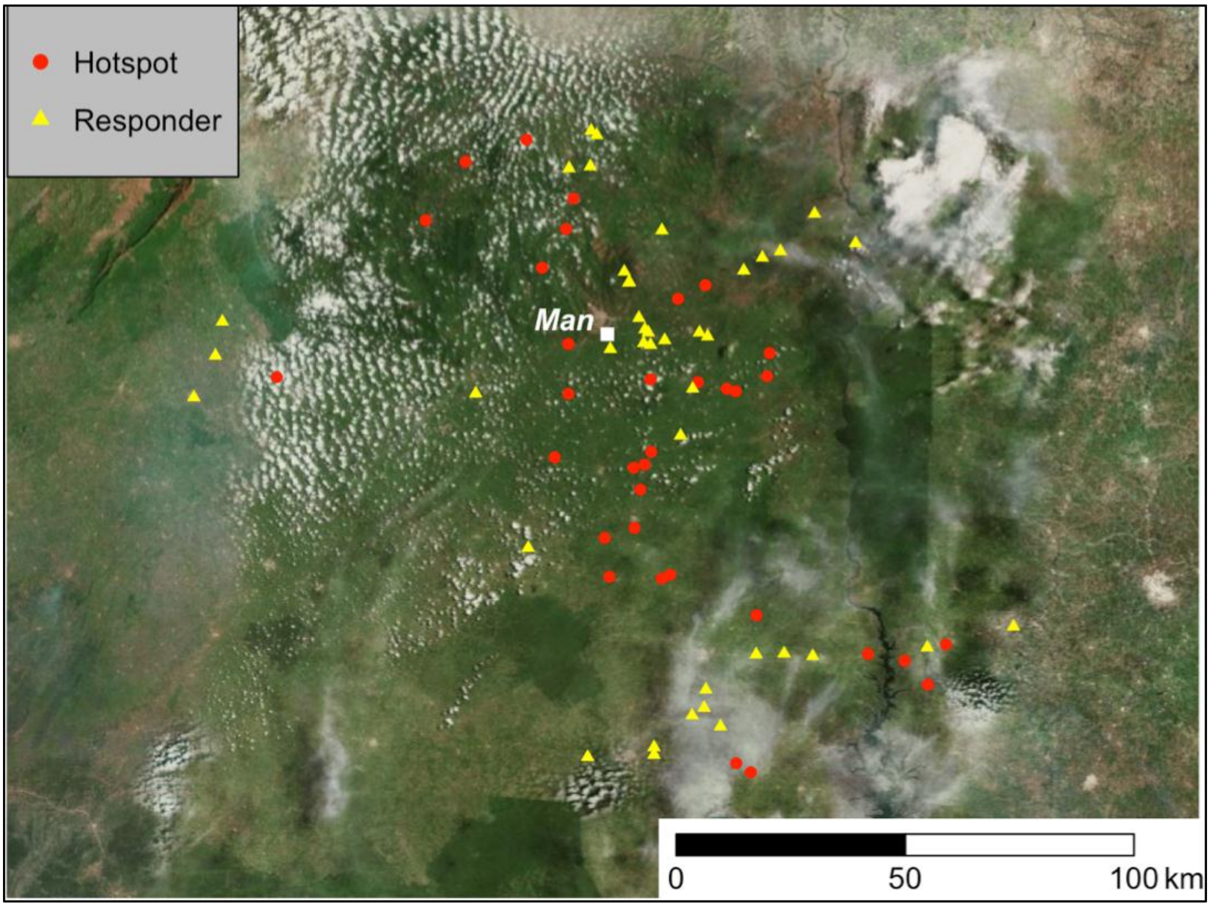

Note: Supplemental Tables 1,2, 3 and 4 will be available online in final publication
